# Influence of aging on the activity of mice Sca-1^+^CD31^−^ cardiac stem cells

**DOI:** 10.18632/oncotarget.13930

**Published:** 2016-12-14

**Authors:** Qiong Wu, Jinxi Zhan, Shiming Pu, Liu Qin, Yun Li, Zuping Zhou

**Affiliations:** ^1^ School of Life Sciences, Guangxi Normal University, Guilin, China; ^2^ Guangxi Universities Key Laboratory of Stem cell and Biopharmaceutical Technology, Guangxi Normal University, Guilin, China; ^3^ Research Center for Biomedical Sciences, Guangxi Normal University, Guilin, China

**Keywords:** cardiac resident stem/progenitor cells, aging, differentiation, proliferation, Gerotarget

## Abstract

Therapeutic application of cardiac resident stem/progenitor cells (CSC/CPCs) is limited due to decline of their regenerative potential with donor age. A variety of studies have shown that the cardiac aging was the problem of the stem cells, but little is known about the impact of age on the subgroups CSC/CPCs, the relationship between subgroups CSC/CPCs ageing and age-related dysfunction. Here, we studied Sca-1+CD31− subgroups of CSCs from younger(2~3months) and older(22~24months) age mice, biological differentiation was realized using specific mediums for 14 days to induce cardiomyocyte, smooth muscle cells or endothelial cells and immunostain analysis of differentiated cell resulting were done. Proliferation and cell cycle were measured by flow cytometry assay, then used microarray to dissect variability from younger and older mice. Although the number of CSCs was higher in older mice, the advanced age significantly reduced the differentiation ability into cardiac cell lineages and the proliferation ability. Transcriptional changes in Sca-1+CD31− subgroups of CSCs during aging are related to Vitamin B6 metabolism, circadian rhythm, Tyrosine metabolism, Complement and coagulation cascades. Taking together these results indicate that Cardiac resident stem/progenitor cells have significant differences in their proliferative, pluripotency and gene profiles and those differences are age depending.

## INTRODUCTION

Aging is an universal phenomenon in living beings, and many geriatric diseases (such as cardiovascular disease and metabolic dysfunction) as results of aging, numerous studies have shown that the stem cell problem primarily results in aging, all ageing phenomena can be interpreted at the level of somatic stem cells [1–3]. Cardiac resident stem/progenitor cells (CSC/CPCs) represent an ideal source of autologous cell-based therapy for heart diseases by maintaining myocardial cell homeostasis. Several populations of CSC/CPCs have been identified based on expression of different stem cell-associated antigens. Sca-1^+^ cells in cardiac tissue may be the most common CSC/CPCs and be relatively easy to isolate [4], Sca-1^+^CD31^−^ cells show cardiomyogenic differentiation and cell transplantation for myocardial repair [5, 6]. Changes in the properties of CSC/CPCs with age involving increased symmetric division, decreased cell proliferation and differentiation, loss of self-renewing capacity, partial depletion of the primitive pool, decreased expression of stem cell markers, impaired the migration ability, and changed the cell cycle into an irreversible quiescent state [7–9]. However, some other reports suggested that the impact of age on the quantity and quality of human cardiac stem cells is quite limited [10]. Clearly, many aspects about stem cells and aging remain to be understood. The influence of age on CSC/CPCs activity is critically important for an organism during development and senescence. We have analyzed the phenotypic, genotypic features and biological-related changes in Sca-1^+^CD31^−^ CSC/CPCs of mice heart from different ages, this will establish a framework for future comparative and functional studies.

## RESULTS

### CSCs isolation and phenotype

**Figure 1:**
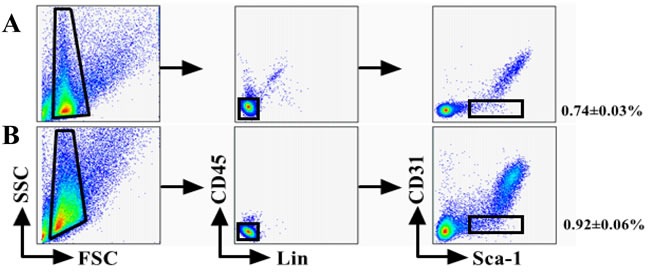

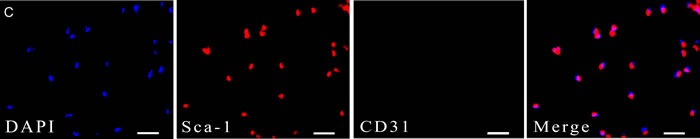
sorting strategy for isolating Lin^**−**^CD45^**−**^ Sca^**−**^1^**+**^CD31^**−**^cells from young (A) and old (B) C57BL/6 mice The percentage of cells was calculated based on a mean of twenty mice. Differences were analyzed with Student's *t*-test (*P* < 0.05). **C.** Analysis of CD31 and Sca-1 expression by IF staining. All cells were triple-stained for Sca-1 (red), CD31 (green), and DAPI (blue). Sca-1 expression in Sca-1^+^CD31^−^ cells with no CD31 expression in Sca-1^+^CD31^−^cells.Merging fluorescent signals showed no heterogenous populations after FACS-based sorting. Scale bars, 10 μm.

We have used FACS to isolate CSCs using Lineage, CD45, Sca-1, and CD31 markers whose expression is conserved across mouse strains and during aging [11]. The Lin^−^CD45^−^Sca-1^+^CD31^−^cells were isolated from younger or older C57BL/6 mice (Figure 1A-B). The percentage of cells was calculated based on a mean of twenty mice, consistent with previous works and the number of CSCs was higher from older mice heart than younger (P < 0.05). Analysis of CD31 and Sca-1 expression by immune fluorescence staining revealed only express Sca-1in CSCs (Figure 1C).

### *In vitro* differentiation of Sca-1^+^CD31^−^ CSCs from younger and older C57BL/6 mice

**Figure 2:**
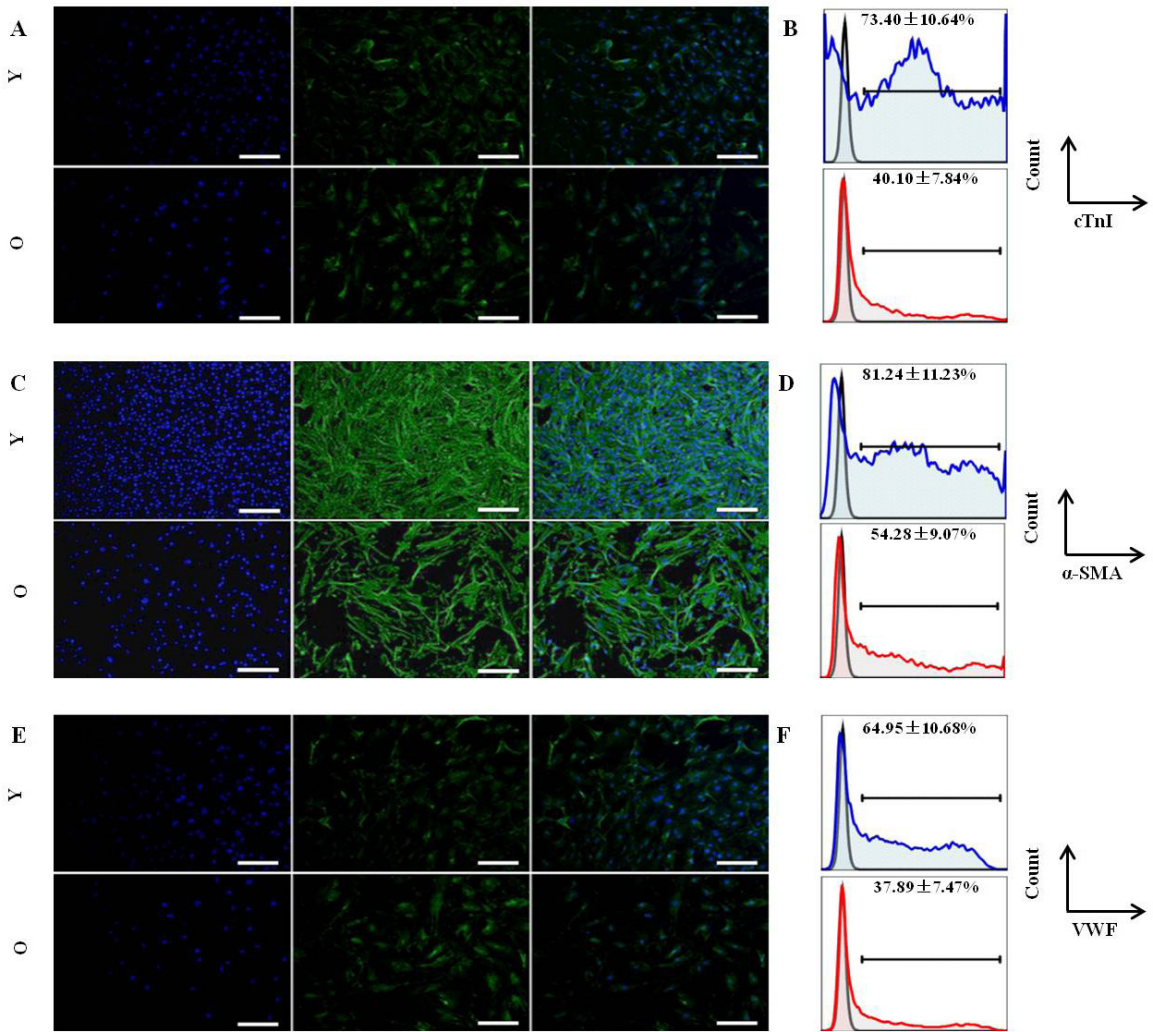
*In vitro* differentiation of CSCs from young and old C57BL/6 mice Cells were stained for specific markers after induction. **A.** Immunofluorescence staining (green) of cardiac-specific marker-cTnI; Nuclei (blue) counterstained with DAPI; then merged images. Y:young mice; O:old mice; **B.** The differentiation efficiency of CSCs for cardiomyocyte from young and old C57BL/6 mice, the value is the percentage of cTnI marker cells among total Sca-1^+^-labeled cells, blue: young mice; red:old mice; **C.** Immunofluorescence staining (green) of smooth muscle marker-α-SMA; Nuclei (blue) counterstained with DAPI; then merged images. Y:young mice; O:old mice; **D.** The differentiation efficiency of CSCs for smooth muscle cells from young and old C57BL/6 mice, the value is the percentage of α-SMA marker cells among total Sca-1^+^-labeled cells, blue: young mice; red:old mice; **E.** Immunofluorescence staining (green) of enodthelial cell marker-VWF; Nuclei (blue) counterstained with DAPI; then merged images. Y:young mice; O:old mice; **F.** The differentiation efficiency of CSCs for endothelial cells from young and old C57BL/6 mice, the value is the percentage of VWF marker cells among total Sca-1^+^-labeled cells, blue: young mice; red:old mice; Every sample was performed in triplicate, differences were analyzed with Student's t-test (*P* < 0.01). Scale bars, 50 μm.

Differentiation is an important feature of stem and progenitor cells. To assess the differentiation activity of CSCs into cardiomyocyte cells, endothelial lineages cells and smooth muscle cells, the same number of Sca-1^+^CD31^−^ cells from younger and older mice were cultured in differentiation medium and control medium (without growth factors). After induction cultivation, specific markers were analyzed by immune fluorescence. After 2 weeks of induction, the cells morphology have changed, Cardiac Troponin I(cTnI)-positive cells, α-Smooth Muscle Actin(α-SMA)-positive cells and Von Willebrand Factor(VWF)-positive cells were detected by IF in younger and older groups (Figure 2-A,C,E). However, the differentiation efficiency results indicated that CSCs from groups of younger had a statistically significant higher (p < 0.01) for cTnI (73.40±10.64% VS 40.10±7.84%), α-SMA(81.24±11.23% VS 54.28±9.07%) and VWF(64.85±10.68% VS 37.89±7.47%) differentiation efficiency compared to older(Figure 2-B,D,F); No Cardiac Troponin I-positive, α-Smooth Muscle Actin-positive and Von Willebrand Factor-positive cells were observed in control groups (data not shown).

Overall, these data indicated that younger mice Sca-1^+^CD31^−^cells have a stronger ability differentiation into cardiac cell lineages.

### Cell proliferation of CSCs from younger and older C57BL/6 mice

**Figure 3:**
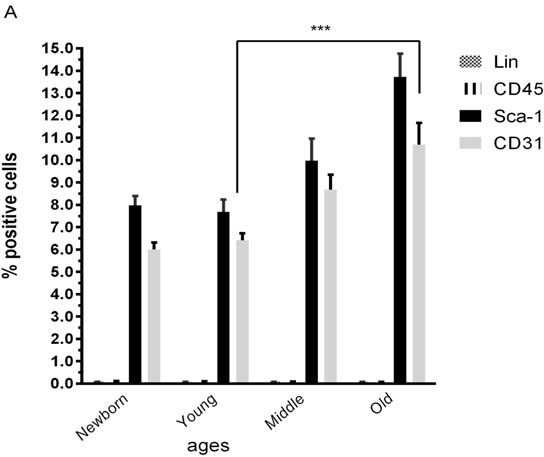

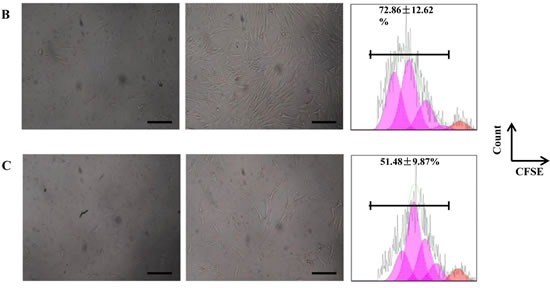
Proliferation profile from mice cardiac stem cells at different age **A.** Characterization by flow cytometry assay of percentage of positives cardiac stem cells marker (Sca-1), endothelial marker (CD31) and hematopoietic markers (Lin and CD45) Newborn(1-3 days), Young(2-3months), Middle(6-8months),Old(22-24months). **B.** Proliferation assay of CSCs from young mice for 6 days. **C.** Proliferation assay of CSCs from old mice for 6 days. One representative experiment is shown. Differences were analyzed with Student's *t*-test (*P* < 0.05). Scale bars, 50 μm.

Characterization of populations of CSCs from different ageing group by flow cytometry reveled that no statistical significant differences exist between group respects levels of stem cell markers Lin and CD45 used for that, positive cells for CD45 and Lin were less 1%, but the significant differences between younger and older group respects for CD31 and Sca-1 (Figure 3A) indicated that the number of CSCs was higher in older mice.

Proliferation assays results indicated that CSCs from groups of younger (72.86 ±12.62%) had a statistically significant higher (p < 0.05) proliferation ability compared to older (51.48 ± 9.87%) (Figure 3B-C).

### Cell cycle of CSCs from younger and older C57BL/6 mice

**Figure 4:**
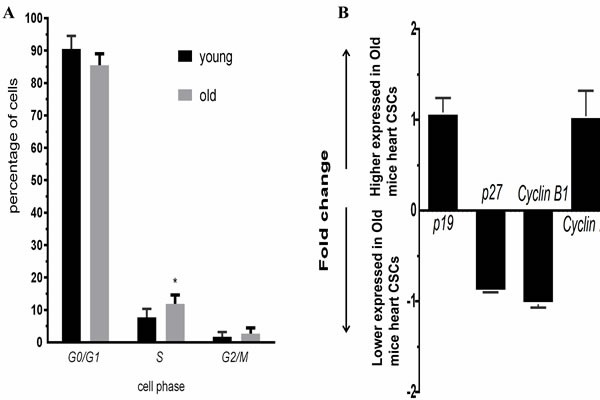
**A.**Cell cycle of CSCs from young and old C57BL/6 mice, blue: young; red:old; **B.** Expression profiles of cell cycle genes detected by QRT-PCR. Each bar represents the fold change of genes expression in old mice heart CSCs cells VS young mice heart CSCs cells. Expression levels were nomalized to that of gene GAPDH and the expression level of young mice heart CSCs cells was used as calibrator to calculate the fold change. Calculated difference change based on a mean of three biological replicates. The ΔΔC_T_ values obtained were subjected to unpaired Student's t test implemented in Prism software. Bars above and below the x-axis show genes that are up- or down-regulated, respectively, in old mice heart CSCs cells. The asterisks indicate samples whose values are statistically significantly, *P < 0.05, **P < 0.01, ***P < 0.001.

The percentage of CSCs in G0/G1, S, and G2/M phase of the cell cycle was evaluated from younger and older mice heart CSC cells. We found that 90.55±4.01% of younger mice heart CSC cells were in G0/G1 phase, 7.72±2.64% in S phase and 1.73±1.41% in G2/M phase; 85.45±3.54% of older mice heart CSC cells were in G0/G1 phase, 11.86±2.71% in S phase and 2.69±1.74% in G2/M phase; the percentage of cell in S phase was significantly increase (p<0.05) of older mice heart CSC cells. Conversely, the percentage of cells in G0/G1 and G2/M phase was no significantly change between older and younger mice (Figure 4A). These changes were associated with the expression of genes involved in cell cycle. No significant differences were observed for the expression of *p19*, *p27*, *Cyclin B1* and *Cyclin D1* between younger and older mice heart CSC cells (Figure 4B).

### MRNA Array Analysis

**Figure 5:**
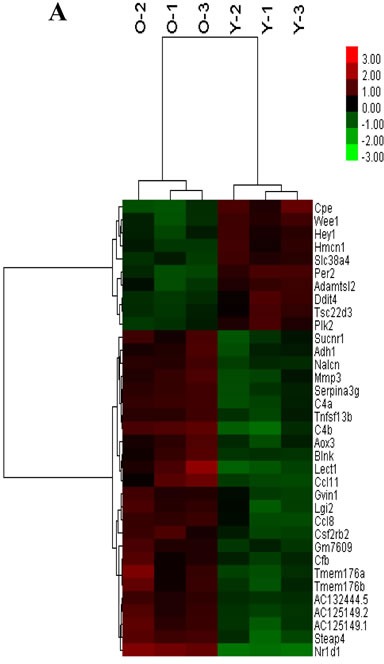

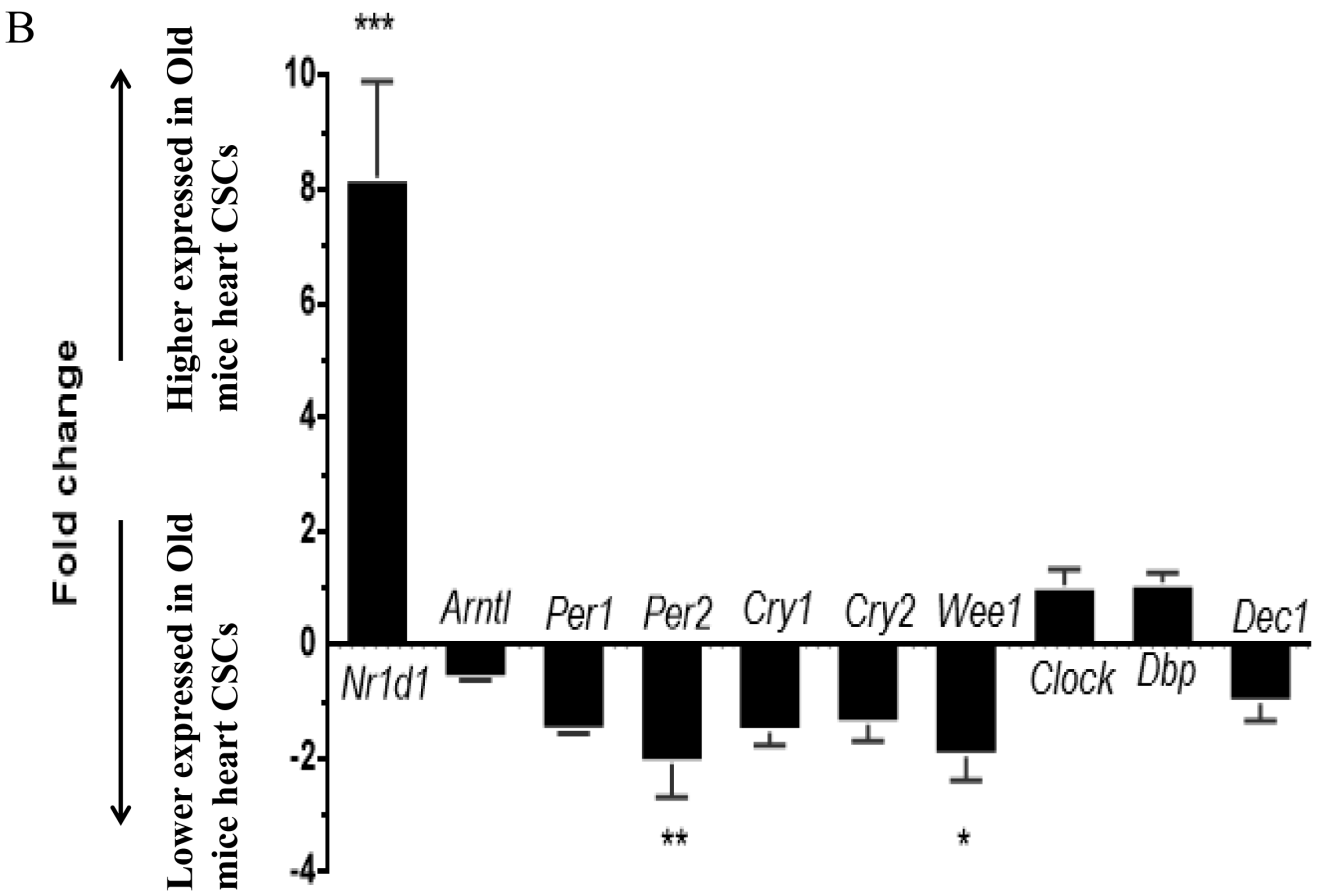

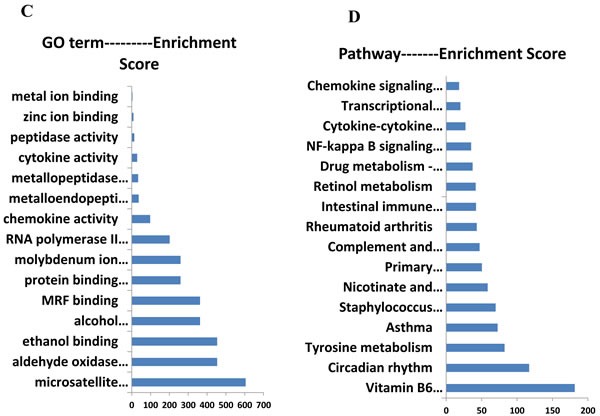
The mRNA expression signatures in old mice CSCs versus young mice CSCs **A.** The tree was based on the log_2_ transformation of the normalized probe signal intensity using hierarchical clustering. O-old mice CSCs; Y-young mice CSCs; Red: up expression genes; Green: down expression genes. Every sample was performed in triplicate. **B.** Expression profiles of circadian clock genes detected by real time RT-PCR. Each bar represents the fold change of genes expression in old mice heart CSCs cells VS young mice heart CSCs cells. Expression levels were nomalized to that of gene GAPDH and the expression level of young mice heart CSCs cells was used as calibrator to calculate the fold change. Calculated difference change based on a mean of three biological replicates. The ΔΔC_T_ values obtained were subjected to unpaired Student's t test implemented in Prism software. Bars above and below the x-axis show genes that are up- or down-regulated, respectively, in old mice heart CSCs cells. The asterisks indicate samples whose values are statistically significantly, *P < 0.05, **P < 0.01, ***P < 0.001.**C.** Functional categories of differentially expressed genes. **D.** Pathway enrichment of differentially expressed genes.

Expression profiling of undifferentiated Sca1^+^CD31^−^ cells from younger or older mice. A total of 35 genes were differentially expressed between younger and older mice (false discovery rate < 0.05 and folder-change ≥ 2, Figure 5A, Table 1). There existed 25 up-regulated and 10 down-regulated mRNAs in older mice when compared to younger. Furthermore, after GO function and KEGG pathway analysis, these differential expression genes were significantly enriched function in microsatellite binding, aldehyde oxidase activity and et al. (Figure 5C); enriched pathways in Vitamin B6 metabolism, circadian rhythm, Tyrosine metabolism, Complement and coagulation cascades and et al (Figure 5D).

**Table 1:** Differentially expressed mRNAs

Gene Symbol	Gene Description	Fold Change	*p*-value	Gene Feature
Cpe	carboxypeptidase E	−3.13329	0.000122	down
Per2	period circadian clock 2	−2.76163	0.000073	down
Wee1	WEE 1 homolog 1	−2.42462	0.00014	down
Slc38a4	solute carrier family 38, member 4	−2.08692	0.000149	down
Hmcn1	hemicentin 1	−2.06809	0.000318	down
Plk2	polo-like kinase 2	−2.0679	0.000327	down
Ddit4	DNA-damage-inducible transcript 4	−2.22285	0.000425	down
Adamtsl2	ADAMTS-like 2	−2.19011	0.000408	down
Hey1	Hairy enhancer-of-split related with YRPW motif 1	−2.00755	0.000452	down
Tsc22d3	TSC22 domain family, member 3	−2.02136	0.000612	down
Nr1d1	nuclear receptor subfamily 1, group D, member 1	6.598208	0.000047	up
C4b	complement component 4B	3.865675	0.000051	up
Lect1	leukocyte cell derived chemotaxin 1	3.839932	0.000252	up
Steap4	STEAP family member 4	3.274248	0.000065	up
Ccl11	chemokine (C-C motif) ligand 11	3.03196	0.000354	up
AC125149.1		2.857723	0.000109	up
C4a	complement component 4A	2.596466	0.000078	up
AC125149.2		2.482776	0.000105	up
Mmp3	matrix metallopeptidase 3	2.435376	0.000283	up
Ccl8	chemokine (C-C motif) ligand 8	2.410426	0.000292	up
Serpina3g	serine peptidase inhibitor, clade A,member 3G	2.397298	0.000096	up
Csf2rb2	colony stimulating factor 2 receptor, beta 2, low-affinity (granulocyte-macrophage)	2.396598	0.000225	up
Blnk	B cell linker	2.303156	0.000287	up
Tnfsf13b	tumor necrosis factor (ligand) superfamily, member 13b	2.215785	0.000145	up
Nalcn	sodium leak channel	2.214615	0.000091	up
Gm7609	predicted pseudogene 7609	2.140701	0.000203	up
AC132444.5		2.125757	0.000056	up
Tmem176a	transmembrane protein 176A	2.848028	0.000702	up
Tmem176b	transmembrane protein 176B	2.52496	0.000559	up
Lgi2	leucine-rich repeat LGI family, member 2	2.40872	0.000608	up
Sucnr1	succinate receptor 1	2.330601	0.000639	up
Aox3	aldehyde oxidase 3	2.239808	0.000483	up
Gvin1	GTPase, very large interferon inducible 1	2.060591	0.000519	up
Adh1	alcohol dehydrogenase 1 (class I)	2.08528	0.001018	up
Cfb	complement factor B	2.034334	0.001178	up

The mRNA expression level in old mice CSCs versus young mice CSCs. All genes had *p*-value < 0.05 and fold change ≥2. Down: down expression genes in old mice CSCs; Up: up expression genes in old mice CSCs.

The circadian rhythms genes were significantly changed with aging [12]. To validate the microarray data, 10 circadian clock genes were chosen for analysis by real time RT-PCR: *Per1*,*Per2*,*Cry1*,*Cry2*,*Clock*, *Arntl*, *Nr1d1*,*Dbp,Wee1*and*Dec1*. Among these genes, with higher expression of *Nr1d1* (P<0.001) and lower expression of *Per2* and *Wee1* (P<0.05) in older mice CSCs as compared to younger, and real time RT-PCR results confirmed the mRNA microarray results (Figure 5B).

## DISCUSSION

The characteristics of stem cells such as cell cycle, self-renewability, proliferation and lineage composition are altered with aging [2]. Senescent cells accumulate in tissues with advancing age. Tissue-specific stem cells, for example those of the hematopoietic and musculoskeletal system are also known to undergo degenerative changes with age [13], but impact of age on the quantity and quality of human cardiosphere-derived cells (CDCs, as one of cardiac stem cells subgroups) is quite limited [10]. However, few studies have focused on the influence of donor age on other subgroups of CSC. Here, we tested whether the regulation of stem-cell activity of Sca-1^+^CD31^−^CSCs were impaired as donor age increased. The current study compared Sca-1^+^CD31^−^CSCs from younger (2–3 months) and older (22–24 months) C57BL/6 mice as biological age. Although the number of CSCs was higher in older mice, the advanced age significantly reduced the differentiation ability into cardiac cell lineages (Figure 2) and the proliferation ability (Figure 3B-C). The cell cycle stages G1, S, G2 and M are time-dependent, and the expression of some key cell cycle genes play an important role in the process of the cell cycle changed. The Clock genes such as *Per1*, *Per2*, *Clock* and *Bmal* play an important role in regulating cell proliferation through some cell cycle-related genes, such as *Wee1*, *c-Myc* and *Ccnd1* [14]. The circadian clock affects the cell proliferation through the protein WEE1, to regulate the cell cycle, and the *wee1* expression was directly regulated by the circadian clockwork [15, 16]. In our results, *Wee1* expression with a 2.4 folder change down expression in older mice heart CSC, however no significant differences were observed for the expression of *p19*, *p27*, *Cyclin B1* and *Cyclin D1* between younger and older mice heart CSC cells. *Per2* regulated the cell cycle transition from G1 to S stages [17], the lower expression of *Per2* and *Wee1* (P<0.05) at older mice CSCs as compared to younger mice CSCs related with the percentage of cell in S phase was significantly increase (p<0.05) of older mice heart CSC cells (Figure 4A).

For determining the molecular basis for differences between younger and older Sca-1^+^CD31^−^CSCs in differentiation, proliferation and cell cycle, analysis of mRNA profiling in younger and older Sca-1^+^CD31^−^CSCs. We found that the expression profile showed function in microsatellite binding, aldehyde oxidase activity and et al. The differently expressed genes showed pathway in Vitamin B6 metabolism, circadian rhythm, Tyrosine metabolism, Complement and coagulation cascades et al. The heart as peripheral oscillator for 13% of genes expressed exhibit significant circadian rhythmicity [18]. The circadian clock regulate the biological rhythm through transcription-translation feedback [19]. As a clock gene, *Rev-erbα* focused more on its

effects on adipogenesis and as a key mediator between clockwork and inflammation [20]. *Rev-erbα* expression gradually decreased during BMSC osteogenesis, and overexpression of *Rev-erbα* in BMSCs inhibited cell proliferation and osteogenesis, suggested age-related BMSC changes may be associated with the expression of *Rev-erba* [21]. Some research compared the *Rev-erbα* expression levels in different tissues of rats at the ages of 1 month, 3 months, and 6 months, the results showed a decreasing trend during aging, suggested *Rev-erbα* levels may be associated with tissue aging [22]. However, in our results, the *Rev-erbα* expression was higher in older mice heart CSC (Table 1; Figure 5), the increasing *Rev-erbα* levels may play an important role in activity of CSC during aging. Among clock genes,*Per2* as mediator of vascular senescence, immune response, angiogenesis and endothelial function,plays an important role in cell cycle progression, the balance of cell proliferation and apoptosis [23], the pathophysiology of the cardiovascular [24] and aging [25]. Some reports showed that no significant differences in the rhythm of expression of*Per2*and*Bmal1*, as well as that of*Dbp*was found in the heart of aged*vs*younger mice [26]. However, in our results, the *Per2*expression was lower in olderer mice heart CSC, may be relative with the decreasing differentiation ability of older mice CSC. *Per1* and *Per2* expression pattern with aging in other reports [12] was consistent with our results. With aging, we suggested differently express in several rhythmic genes, with the effects somewhat relative with CSC differentiation. The reason for this difference is not clear. We next sought to research the biological significant circadian clock mechanism in CSCs. Thus, differences about this clock genes expression over CSCs on aging may reflect distinct functions.

The functions of the immune system also altered with aging, so that it no longer resemble the immune system of the younger individuals [27]. The complement system played an important role in both innate and acquired immunogenicity. The complement pathway has been implicated in the pathogenesis of many age-related diseases, e.g macular degeneration [28], arrhythmogenic cardiomyopathy (ACM) [29, 30]. The complement component C4 which has the isotypes C4A and C4B is an effector protein of the immune system [31], the C4A protein is more relevant in immunoclearance, whereas C4B is more important in the defense against microbes [32]. Expression of complement component C1q, C3, C4, C5 and factor B up expressed with aging in C57BL/6 mice [33]. Inhibiting C5aR (CD88) signaling improves cardiac function, suggested that complement modulation could be a new therapeutic strategy for ACM [29, 30]. In our results, the expression of complement component C4a, C4b and inflammation genes Ccl11, Ccl8 were up expressed in older CSCs, indicated that targeting the complement system for the management of cardiac disease maybe a new therapy tool.

As the number of CSCs was higher in older mice, but the older mice Sca-1^+^CD31^−^cells have weaker differentiation ability into cardiac cell lineages and weaker proliferation ability *in vitro*. The gene profile findings indicate that the molecular clockwork is mostly preserved in the aged heart. These findings suggest that *Rev-erbα*, *Per2* and *Wee1* are directly or indirectly affected the circadian rhythm-dependent changes in aging-induced CSC properties. However, the mechanism for a circadian rhythm-dependent change in *Rev-erbα*, *Per2* and *Wee1* expression by aging remains to be determined. The next objective of our work was to investigate whether these differently expressed circadian rhythm genes control CSC differentiation capacities, regulate cell cycle, and play a role in cell proliferation abilities with aging.

## CONCLUSIONS

The stem-cell activity of CSCs assessed by cell proliferation, cell cycle and cell differentiation ability varied among CSCs and was obviously associated with donor ages. This suggests that CSCs from older mice are more prone to undergo senescence than those isolated from younger and age is a critical determinant of the activity of CSCs. The importance of our study lies in the fact that age from the CSCs source directly influences their differentiation, proliferative and gene profiles, also it is the first time where is shown the direct molecular difference of circadian rhythm genes *Rev-erbα*, *Per2* and *Wee1*, immune response genes *C4b*, *Ccl11*, and *Cfb* on participated in aging of CSCs. Summary we affirm that younger group of mice has the most proliferative and pluripotent CSCs be able to future functional studies.

## MATERIALS AND METHODS

### Experimental animals and ethics statement

Younger(2~3months) and older(22~24months)C57BL/6J mice (Experimental Animal Center of Guilin Medical College, China) were used for cell isolation. All procedures involving animals were approved by the Institutional Animal Care and Use Committee.

### Cell isolation and fluorescence-activated cell sorting

Whole hearts were extracted from either male or female of younger and older C57BL/6J mice, split, and washed several times with ice-colder PBS to remove residual blood, and then incubated with indicated combinations of monoclonal antibodies against mouse antigens Sca-1-PE-Cy7, CD31-APC, CD45-FITC, Lin-PE (eBioscience) or with isotype-matched control antibodies before analysis by fluorescence-activated cell sorting (FACS). Freshly sorted CSC (Lin^−^CD45^−^Sca-1^+^CD31^−^) were used for experiments, except as indicated. The methods were expanded as described [34].

### Cell culture and *in vitro* differentiation

The same number of Lin^−^CD45^−^Sca-1^+^CD31^−^cells from younger or older mice heart were initially seeded in normal medium. For cardiomyocyte, smooth muscle cell and endothelial cell induction, cells were cultured in DMEM/F12 supplemented with growth factors for 14 days. The methods were expanded as described [34].When induction was complete, lineage-specific markers were analyzed by IF staining.

The differentiation efficiency was applied to indicate the differentiation ability of Sca-1^+^CD31^−^cells from younger or older mice heart, and the value is the percentage of lineage-specific marker cells among total Sca-1^+^-labeled cells, and was assessed using FACSverse (BD Biosciences).

### The cells *in vitro* proliferation

Cells proliferation assays were carried out as described previously [35].The cells proliferation was assessed using FACSverse (BD Biosciences). Proliferation index was applied to indicate the proliferative status of Sca-1^+^CD31^−^cells from younger or older mice heart, and the value (percentage of CFSE-diluted cells among total CFSE-labeled CSC cells) was designed as 1 for cultures with medium only, and those for other culture conditions were normalized to the medium only group.

### Cell cycle analysis

Cells cycle analysis were using Propidium Iodide Flow Cytometry Assay Kit (Abcam, USA), Briefly, about 1 × 10^5^ cells/ml of CSC cells from younger and older mice were seeded into 24 well plates and kept under 37°C, 5% CO_2_overnight. Fixation of the cells was carried out using 66% ethanol for at least 2 h at 4°C. After centrifugation at 500 × *g* for 5 min, the cells were washed with PBS prior to staining. About 200 μl of staining solution composed of 1 × PI and RNase was added to the cell suspension and incubated in dark at 37°C for 20–30 min. The signals of at least 1×10^4^single cells were recorded on a FACS Canto II flow cytometer (BD Biosciences, Heidelberg, Germany), and DNA histograms were analyzed with the WinCycle software (Phoenix Flow Systems, San Diego, CA, USA) after doublet exclusion.

### RNA preparation and quality control

Total cellular RNA was extracted from 1× 10^5^ Lin^−^CD45^−^Sca-1^+^ CD31^−^ cells from younger or older mice heart, for three samples, isolated using FACS, 10 mice as a pool for per sample. RNA was extracted using TRIzol reagent (Invitrogen) according to the manufacturer's instructions. RNA concentrations were determined photometrically using a NanoDrop 1000 (Peqlab, Erlangen, Germany). Overall RNA quality was assessed by 1% agarose gels with 1 kb molecular weight marker separated in parallel.

### Quantitative real-time RT-PCR

Total RNA was isolated and 1 μg of it was reverse transcribed with oligo dT primers using a first strand cDNA synthesis kit (Promega) according to manufacturer's instructions. cDNAs were PCR amplified (40 cycles at 95°C for 10 s, 60°C for 30 s and 72°C for 30 s) using SYBR green master mix (Takara) in 96 wells, followed by a melting curve analysis. Fluorescence intensities were analyzed in real time ABI/Prism 7700 Sequences Detector System (Applied Biosystems), and the relative quantitation (ΔΔCt) method was used to evaluate gene expression between the younger and older mice CSC cells for each gene examined. The expressions of the genes were normalized to a housekeeping gene, GAPDH.

### Whole genome gene expression microarrays and Bioinformatical analysis of data

The methods were expanded as described [34].

### Immune fluorescence (IF)

The methods were expanded as described [34].

### Statistical analysis

Signal intensity and mRNA expression levels were analyzed by Student's *t*-test or one-wayANOVA. Data are expressed as mean±standard deviations for each parameter measured in each group. Statistical analyses for QRT-PCR were performed with GraphPad Prism 6 software (GraphPad software, CA, USA). Unpaired t-test was used for two-groups comparisons, Two sided p-value was calculated. P<0.05 was considered statistically significant.
